# Postrelease movement and habitat selection of translocated pine martens *Martes martes*

**DOI:** 10.1002/ece3.6265

**Published:** 2020-05-14

**Authors:** Catherine M. McNicol, David Bavin, Stuart Bearhop, Josie Bridges, Elizabeth Croose, Robin Gill, Cecily E. D. Goodwin, John Lewis, Jenny MacPherson, Daniel Padfield, Henry Schofield, Matthew J. Silk, Alexandra J. Tomlinson, Robbie A. McDonald

**Affiliations:** ^1^ Environment and Sustainability Institute University of Exeter Penryn UK; ^2^ Vincent Wildlife Trust Eastnor Ledbury UK; ^3^ Centre for Ecology and Conservation University of Exeter Penryn UK; ^4^ Forest Research Alice Holt Lodge Farnham UK; ^5^ Wildlife Vets International Keighley UK

**Keywords:** carnivore, conservation, reinforcement, reintroduction, restoration, rewilding, translocation

## Abstract

Monitoring postrelease establishment and movement of animals is important in evaluating conservation translocations. We translocated 39 wild pine martens *Martes martes* (19 females, 20 males) from Scotland to Wales. We released them into forested areas with no conspecifics in 2015, followed by a second release in 2016, alongside the previously released animals. We used radio‐tracking to describe postrelease movement and habitat selection. Six martens (15%) were not re‐encountered during the tracking period, of which four undertook long‐distance dispersal. For the remaining individuals, we characterized two phases of movement, “exploration” followed by “settlement,” that differed between releases. In the first release, martens remained in exploration phase for a mean of 14.5 days (*SE* = 3.9 days) and settled at a mean distance of 8.7 km (*SE* = 1.8 km) from release sites, whereas martens released in year two, alongside resident conspecifics, traveled away from release sites at a faster rate, settling sooner, at a mean of 6.6 days (*SE* = 1.8 days), but further, at a mean distance of 14.0 km (*SE* = 1.7 km) from release sites. Animals released in year one did not exhibit habitat preferences overall but within forests they favored recently felled areas, whereas animals released in year two showed strong selection for forested habitat but did not discriminate between forest types. The presence of conspecifics appeared influential for settlement and site fidelity of translocated martens and was associated with more rapid but more distant dispersal of the later cohort. Releases of animals in close proximity appeared to promote site fidelity and rapid establishment of ranges in the recipient environment.

## Graphical abstract

We translocated 39 wild pine martens *Martes martes* (19 females, 20 males) from Scotland to Wales. We released them into forested areas with no conspecifics in 2015, with a second release in 2016, alongside previously released animals. The presence of conspecifics appeared influential for settlement and site fidelity of translocated martens but was associated with more distant and more rapid dispersal of the later cohort. Releases of animals in close proximity appeared to promote site fidelity and rapid establishment of ranges in the recipient environment.

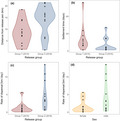

## 1 INTRODUCTION

Translocation, the deliberate movement of organisms from one site to release in another (IUCN, 2013), is a long‐established and frequently used tool in species conservation. In recent years, conservation translocations have increasingly been associated with restoration ecology (Seddon, 2010) as well as being effectively implemented in threatened species recovery projects (Hayward et al., 2007). The return of species to their historic ranges can benefit not only the species in question, but improve functionality and biodiversity within the recipient ecosystem (Seddon, 2010). Successful reintroductions require a sound knowledge of the species’ ecology within its native range as well as some insight into its likely postrelease behavior and habitat requirements. Understanding postrelease movement, habitat selection and the drivers of these is therefore necessary for appraising and improving current and future translocation projects.

Two key components of the success of translocation and reintroduction projects are release site fidelity and the survival rate of the translocated individuals (Armstrong & Seddon, 2008). The selection of appropriate habitat for release sites and, thereby, providing access to adequate resources for individual animals, is paramount for their retention on, or near, the release site (Armstrong & Seddon, 2008). Alongside site characteristics, sex ratio, release schedule, and numerous other factors can influence the likelihood of a new population establishing successfully (Armstrong & Seddon, 2008; Letty, Marchandeau, & Aubineau, 2007). Lack of site fidelity is clearly unfavorable and often implies poor selection of release sites, inappropriate release protocols, and/or unforeseen conspecific interactions (Letty et al., 2007). Therefore, understanding the patterns of movement of translocated individuals after their release, and the characteristics of their selected habitats during the initial release period, is vital in evaluating and improving conservation translocations (Armstrong et al., 2013).

The first individuals to be released into a new area may be more likely to leave the vicinity of release sites, due to the inherent absence of resident conspecifics and/or lack of mating opportunities (Mihoub, Robert, Gouar, & Sarrazin, 2011). Exploration by the introduced animals of the novel environment in search of ideal habitat is a central but unpredictable part of a reintroduction project (Armstrong et al., 2013). Such exploratory movements by translocated individuals can be detrimental to survival, since extended periods of exploration and habitat searching are often erratic and extend over long distances, making them energetically costly (Robertson & Harris, 1995; Spinola, Serfass, & Brooks, 2018; Yott, Rosatte, Schaefer, Hamr, & Fryxell, 2011) and exposing animals to diverse hazards. Three major postrelease movement patterns have been identified: (1) immediate settlement, (2) dispersal followed by settlement, and (3) long‐distance dispersal or failure to settle (Broquet et al., 2006; Davis, 1983; Slough, 1989; Tolhurst, Grogan, Hughes, & Scott, 2015; Woodford, Macfarland, & Worland, 2013). Among translocated carnivores, these patterns have been described in American marten *Martes americana* (Davis, 1983; Slough, 1989; Woodford et al., 2013), otters *Lontra canadensis* (Sjoasen, 1997; Spinola et al., 2018), red foxes *Vulpes vulpes* (Tolhurst et al., 2015), and swift foxes *Vulpes velox* (Moehrenschlager & Macdonald, 2003). However, explanations for the ecological mechanisms driving among‐animal variation in the observed patterns remain ambiguous, with conspecific attraction, habitat suitability, and predation risk all thought to play a role (Davis, 1983; Letty et al., 2007; Sjoasen, 1997). Reduction of problems arising from exploration, long‐distance dispersal, or attempted “homing” has most commonly been achieved through adopting a soft‐release protocol, allowing acclimatization of individuals to the release site in an enclosure provisioned with food for a short period of time prior to release (Moehrenschlager & Macdonald, 2003; Tolhurst et al., 2015).

The presence of conspecifics may be beneficial at low densities, and founding individuals might discriminate less between habitat types and instead favor proximity to other founder members and the establishment of a “neighborhood” (Shier & Swaisgood, 2011; Stamps, 2001; Ydenberg, Giraldeau, & Falls, 1988). Alternatively, founding individuals might intuitively be expected to select the highest quality locations in an uninhabited landscape, in line with an ideal free distribution (Fretwell & Lucas, 1962; Stamps, 2001). With a continual influx of translocated animals, however, competition would be expected gradually to increase (Stamps, 2001; Stamps & Krishnan, 2005), perhaps leading later arrivals to disperse away from otherwise ideal release sites (Selonen & Hanski, 2007; Stamps & Swaisgood, 2007). Therefore, the social structure of the species as well as the habitat, site, and landscape characteristics must be key considerations in translocation project design and implementation.

Reintroductions have been proposed and implemented as measures to combat the decline of carnivores worldwide. In Great Britain, several native mammalian carnivores have experienced historic declines as a result of predator control, environmental contaminants, deforestation, and demand for fur (Sainsbury et al., 2019). Current efforts are being made to reverse these declines. Since the mid‐20th century, considerable recoveries in the ranges and populations of otter *Lutra lutra*, polecat *Mustela putorius,* badger *Meles meles,* and pine martens *Martes martes* have arisen largely through combinations of increased legal protection, changed control practices, reduction in pollution, and habitat enhancement (Sainsbury et al., 2019). Translocations have also played a role in these species’ recoveries with releases of captive‐bred, wild‐caught, or escaped individuals (Sainsbury et al., 2019). The recovery of British otter populations was accelerated by captive breeding and release of otters (Jefferies, Wayre, & Jessop, 1986). Polecats have also benefited from reintroduction, primarily through illicit releases such as those in Cumbria and Argyll (Birks & Kitchener, 1999). The pine marten is currently showing natural range extension in Scotland (Sainsbury et al., 2019), though its expansion has also been aided by translocation to southern Scotland (Shaw & Livingstone, 1992), and there have likely been sporadic illicit releases in England (Birks & Messenger, 2010; Jordan et al., 2012).

Recovery of the pine marten throughout the UK has been an area of focus for statutory (Bright & Smithson, 1997) and nongovernmental organizations (MacPherson, 2014), with an aim to expand the range extent of what was a sparse and fragmented population through translocations and population reinforcements. Previous translocation studies of *Martes* species have indicated strong site affinity by released individuals (Davis, 1983; Shaw & Livingstone, 1992; Slough, 1989; Woodford et al., 2013). This may partly have been related to the use of soft releases (Davis, 1983; Woodford et al., 2013). Martens are, however, highly mobile animals and are capable of dispersing large distances (Broquet et al., 2006). Long‐distance postrelease movements have been attributed to territorial saturation or the absence of suitable habitat near to release sites (Woodford et al., 2013). In some instances, male martens have been found to disperse further than females (Slough, 1989). This is likely related to sexual dimorphism with regard to body size, energetic demands of reproduction, and ranging extent (Caryl, Quine, & Park, 2012; Zalewski, 2007), as well as pronounced intra‐sexual territoriality, allowing for the overlap of male and female ranges, but exclusivity of ranges within each sex (Powell, 1979; Erlinge & Sandell, 1986; Balharry, 1993; but see Bartolommei, Manzo, & Cozzolino, 2016). These studies also found that although many translocated individuals settled in mature forest, their movement was not impeded by landscape features or the presence of different habitat types (Slough, 1989).

Martens are predominantly viewed as forest specialists (Balestrieri et al., 2010; Balharry, 1993; Bartolommei et al., 2016; Manzo, Bartolommei, Rowcliffe, & Cozzolino, 2012; Slough, 1989; Storch, 1990; Weber, Roth, Tesini, & Thiel, 2018) and often den in tree cavities found in ancient woodland. Nevertheless, martens can traverse and utilize areas of scrub and low canopy cover (Balestrieri et al., 2010; Lombardini et al., 2015; Manzo et al., 2012; Moll et al., 2016; Pereboom et al., 2008), with some studies even showing preferential use of such open habitats (Manzo et al., 2018). In many regions with fragmented forest, pine marten diet is dominated by grassland voles *Microtus* spp., found in edge and open habitats containing tussock grass (Caryl et al., 2012; Hansson, 1978). This contrasts with studies in highly forested regions which have identified the greater importance of forest‐dwelling voles *Myodes* spp. Although mature forest provides the structural complexity required for marten denning and foraging (Caryl, 2008), varied habitat use is linked not only to the level of forest fragmentation but also prey availability and conspecific density (Caryl et al., 2012; Lombardini et al., 2015; Powell, 1979). This suggests that martens are capable of exploiting both forest interiors and the edge habitats abundant in mosaic habitat structure. However, few of these studies have looked at marten movement and habitat selection after a translocation event.

Mid‐Wales was identified as the optimal location for a species recovery program (MacPherson, 2014) to facilitate the spread of pine martens throughout Wales and into England due to its high availability of forested habitats and low‐density road network. Scat surveys undertaken between 2011 and 2015 found no evidence of marten presence in this part of the species’ historic range, suggesting the former resident population was, at best, functionally extinct in the region. Our study examined the movements and habitat use of translocated pine martens immediately after their release. We tracked two cohorts of martens taken from the wild in their core range in Scotland and released in an unoccupied region of their historic distribution in mid‐Wales. First, we describe the initial postrelease movements of martens, characterizing phases of exploration and settlement in years with and without resident conspecifics in a novel environment. Second, we investigate habitat selection by individual martens across a large and diverse habitat matrix and within wooded areas, again in the absence (year 1) and later presence (year 2) of conspecifics. The results of our study improve understanding of marten habitat requirements and post‐translocation movement ecology in unoccupied areas of their historic range. This can be used to inform and maximize the success of future reintroduction programs and to understand the movement ecology of a recovering and expanding population.

## 2 METHODS

### 2.1 Trapping, translocation, and release

**FIGURE 1:**
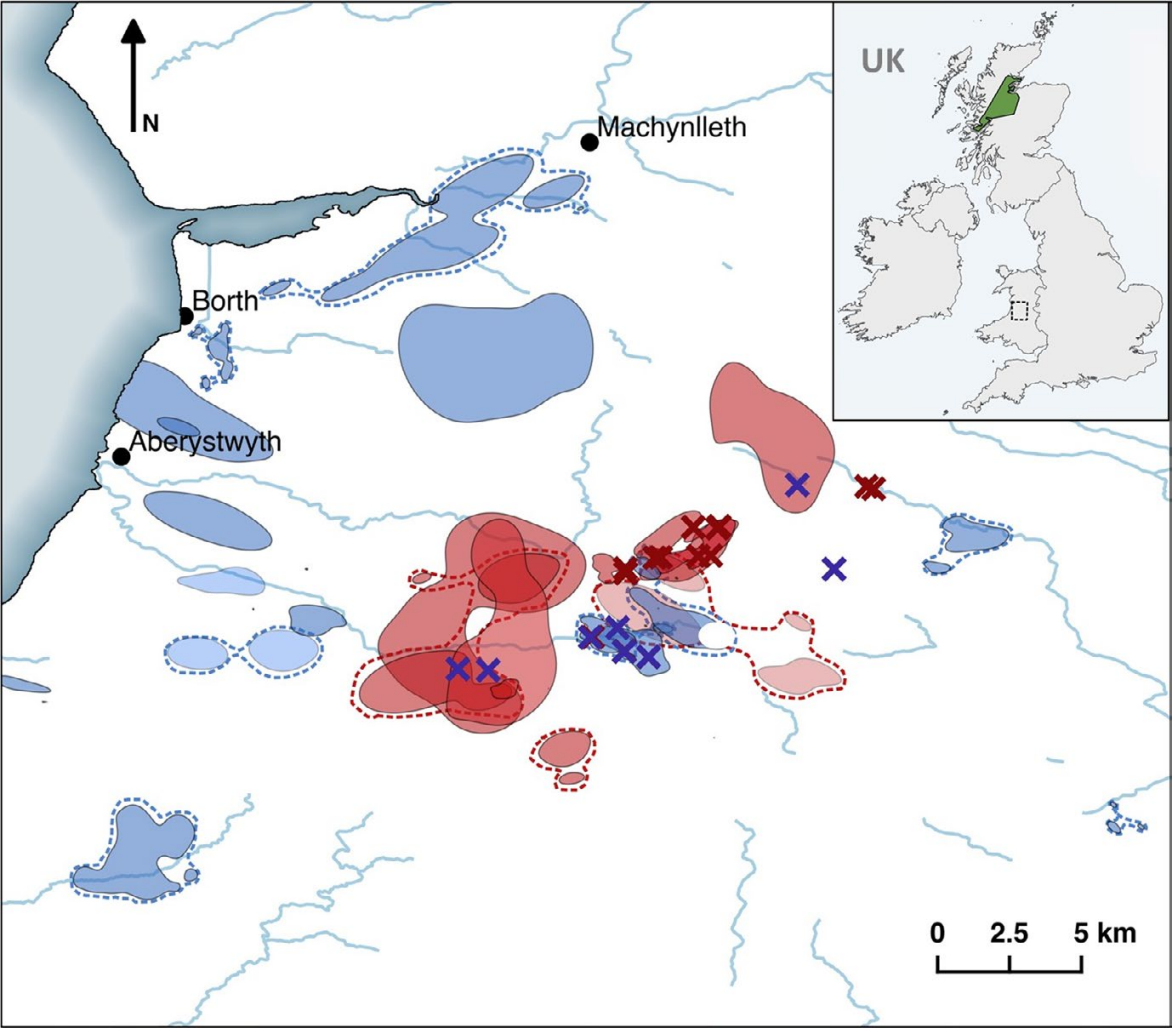
Map of home ranges of translocated pine martens *Martes martes* released in 2015 (red) and 2016 (blue) in Wales. Home ranges are 90% kernel density estimates. Individuals with multiple home range centers are grouped with dotted lines. Release pens, indicated by X, are shown for 2015 (red) and 2016 (blue). Rivers are indicated in blue. Inset map of the UK indicates the region containing trapping locations in Scotland (green) and the release site area in Wales (dashed box)

Between September and November, in both 2015 and 2016, pine martens were translocated from forests in the Scottish Highlands to mid‐Wales (Figure 1). Source sites in Scotland were surveyed for marten scats before live‐capture traps (Tomahawk 205, Tomahawk Live Trap, Hazelhurst, USA) were installed and prebaited for 2–3 weeks. Sites comprised large blocks of commercial conifer plantation with heather *Calluna vulgaris* and bilberry *Vaccinium myrtillus* understorey. Traps were prebaited with peanuts, jam, raisins, raw whole chicken eggs, and scent lure (Blackie's Magnum Call Lure) and then monitored for marten activity by motion‐sensitive trail cameras (Bushnell NatureView HD, Bushnell Corp.) before being set for one night per week until 2–4 individuals per woodland had been caught. This reduced the chance of translocating related individuals and unsustainably depleting resident populations. Trapped animals were processed at a nearby temporary field station. Martens were restrained gently using “combs” inserted between cage bars, in low‐light and quiet conditions. General anesthesia was induced by intramuscular injection of a combination of ketamine (25 mg/kg) and midazolam (0.2 mg/kg). If required during the sampling and collaring period, face‐mask inhalation anesthesia with oxygen and isoflurane was utilized. Animals were given a full health screening by a wildlife veterinarian. Adult martens in good physical condition, at an equal ratio of males to females, were selected for translocation. Any juveniles, surplus individuals, those with any obvious injuries or deemed too old (on the basis of their dentition), were rereleased at their site of capture. Individuals to be translocated were tagged with a subcutaneous passive integrated transponder (PIT: Avid Identification Systems Inc.) and fitted with a collar equipped with a VHF transmitter (Biotrack Ltd.).

Martens were translocated overnight from Scotland to sites in mid‐Wales in a purpose‐modified dog transport van with climate control and physical separation between animals and between animals and drivers. The release sites had been identified in a feasibility study for reintroduction to England and Wales as having high habitat suitability, based on habitat composition, road density, potential impact on other species, and public perceptions (MacPherson, 2014). These sites were dominated by commercial conifer plantations managed on a short rotation, clear‐fell regime and were deemed to present a low‐mortality risk to released animals. The forest was dominated by Sitka spruce *Picea sitchensis* with varying proportions of Norway spruce *Picea abies*, Douglas fir *Pseudotsuga menziesii*, larch *Larix kaempferi, L. eurolepis,* and lodgepole pine *Pinus contorta*. Deciduous and mixed woodland within and surrounding these sites is characterized by small proportions of these conifers alongside sessile oak *Quercus petraea*, beech *Fagus sylvatica*, rowan *Sorbus aucuparia*, birch *Betula* spp., and willow *Salix* spp. Other mesocarnivores, including red foxes, were present in the release areas, as were low numbers of polecats, stoats *Mustela erminea*, weasels *M. nivalis,* and otters (MacPherson, 2014; Sainsbury et al., 2019).

The translocated martens were placed in individual soft‐release pens (timber frames 3.6 × 2.3 × 2.4 m with 16 gauge 24 × 13 mm weldmesh) furnished with vegetation and containing a den box. Animals were held in these pens for 5 days and supplied with food (day‐old chicks, raw eggs, peanut butter, and raisins) *ad lib*. Release was subjected to confirmation, from serological testing of samples taken at the time of capture in Scotland, that individuals had not been exposed to canine distemper virus, following which, the pen door was then opened and animals were allowed to leave. Upon removal of soft‐release pens, a den box was installed nearby (and remained in situ beyond the lifespan of the project) and food was provided until the martens ceased to return (0–3 weeks). Trapping and release protocols in year 1 and year 2 were consistent.

Telemetry locations of pine martens were collected for up to 10 months postrelease with each marten being located at least once per week. Tracking was predominantly undertaken at dusk and after sunset to ensure locations were representative of marten movement during their active hours (Mccann, Zollner, & Gilbert, 2017; Zalewski, 1997). Animals released in 2015 (year 1) were not monitored after the releases undertaken in 2016 (year 2) as VHF collars were removed 6–10 months after their release, prior to battery exhaustion to enable location for retrapping outside of key breeding events. Animals were retrapped under license using live‐capture traps and restrained in a handling cone to enable collars to be cut off. Pine marten locations were triangulated from two locations and bearings taken within 5–10 min of each other (mean = 5 min, *SD* = 4 min, range = 0–57 min) using LOAS 4.0 (Ecological Software Solutions LLC; *n = *1,413, mean = 37 per individual, range = 1–110; Appendix S3). Single bearings that were taken over one hour apart, or did not converge to give a triangulated location, were excluded from the final dataset. To estimate the error of VHF triangulated locations in relation to true collar locations, two observers, who undertook all radio‐tracking, took simultaneous bearings on collars in unknown locations (*n = *14). These points were triangulated, and the distance (m) of the triangulated location from the true collar location was then measured. The median error of VHF locations was estimated as 70 m.

### 2.2 Postrelease movements

For each individual, we calculated the straight‐line distance (km) from the release pen to each triangulated location and modeled these with time since release from pen, measured in days, as a predictor. We fitted a piecewise (“broken‐stick”) linear regression model (Toms & Lesperance, 2003) forced through the origin, representing a period of exploration, followed by settlement. The piecewise regression model was constrained to fit two segmented linear relationships with one intersection point (breakpoint), taken as the point at which settlement took place. The time to settlement (*t*) in days (i.e., where the breakpoint lies on the x‐axis), distance to settlement (*d*) in km (i.e., where the breakpoint lies on the y‐axis), and the rate of dispersal (*r*), in km/day (i.e., the slope of the initial exploration period from the origin to the breakpoint), were treated as parameters of postrelease behavior. As model convergence of piecewise regression can be sensitive to the start parameters and number of iterations, the model fitting was attempted up to 1,000 times, with the first successful fit being extracted. The fit of the piecewise model was compared to that of a simpler linear least squares model of distance and time since release using Akaike's information criterion, adjusted for sample size (AICc [Burnham & Anderson, 2004]). In six cases, a piecewise model could not be fitted due to sparsity of data in the earliest stages following release (i.e., animals went missing for a number of days before being located for the first time), which caused problems with model fit. These individuals were excluded from further postrelease movement analyses.

An individual was considered to be “settled” if the distance moved from their release pen reached a plateau (i.e., the slope of the second line was not significantly different from zero). Before analyses of the postrelease movement parameters, we confirmed there was no correlation between the distance (*d*) and the number of days since release (*t*) at which martens moved from the transition into the establishment phase (Pearson's correlation; ρ = 0.21, t = 1.00, *df* = 21, *p* = .32). Piecewise regressions were fitted using the *R* package *segmented* (Muggeo, 2017), and all analyses were undertaken in *R* version 3.3.3 (R Core Team 2018).

### 2.3 Analysis of movement

Generalized linear models (GLMs; Marschner, Donoghoe, & Donoghoe, 2018) were used to examine the effect of sex and year of release on the three response variables: time to settlement (t), distance to settlement (d, rounded to whole numbers), and rate of dispersal (r). We did not include an interaction term between sex and year in any of the models due to the small sample size of each sex within each group. Day of settlement (t) was modeled using a negative binomial GLM with a (default) log‐link, distance of settlement (d) was modeled using a Gaussian GLM with a square‐root link, and rate of dispersal (r) was log‐transformed to normalize distribution of residuals. We used backward stepwise elimination to determine the minimum adequate model. Variables were retained at each stage if removing them had a significant effect on model fit, as measured using an ANOVA (α = 0.05). We back‐transformed model estimates from the final model to the original scale to obtain response values using the *R* package *emmeans* (Lenth, Singmann, Love, Buerkner, & Herve, 2019).

**FIGURE 2:**
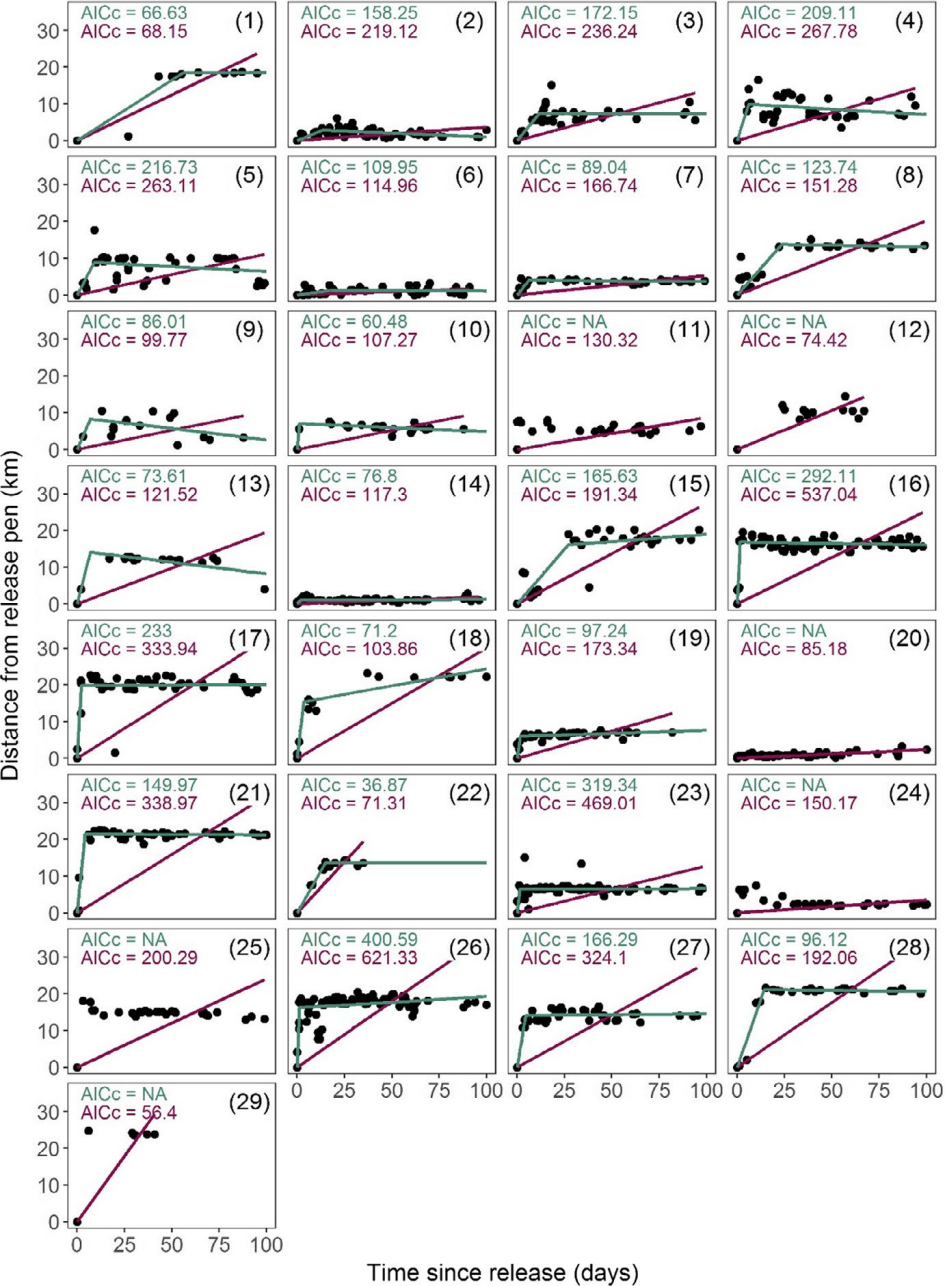
Postrelease movement of translocated pine marten away from release sites over 100 days after release. Each panel represents the movement of an individual marten. The green line shows a “broken‐stick” regression fitted to the data, representing a two‐phase movement pattern. The purple line shows a linear regression fitted to the data representing continuous movement away from the release pen. The AICc values for each model are provided, representing the fit of the data to the model. AICc values enable comparison between broken‐stick and linear models. When AICc = NA, a broken‐stick regression could not be fitted due to scarcity of locations immediately after release. Animal number is shown in parentheses. Animals 1–13 were released in 2015 and 14–29 in 2016

Range size asymptotes were produced prior to generation of home range kernels to ensure ranging data were only generated using individuals with adequate relocation data and stable range sizes. Asymptotes with 95% confidence intervals (CIs) were calculated using an increasing number of resampled locations (Laver & Kelly, 2008) taken after the breakpoint only, up to 100 days postrelease (i.e., during the “settlement” phase). All individuals were initially included in this analysis (*n = *29), including those for which a segmented model (and thus breakpoint) could not be fitted (*n = *6). For these 6 martens, linear model plots were visually inspected and a breakpoint of zero days was assigned, therefore including all of the locations recorded (Figure 2). Individuals (*n* = 3) with an inadequate number of relocations were excluded from calculation of ranging metrics. Home ranges of remaining individuals (from the breakpoint until 100 days postrelease; *n = *26) were then characterized by 90% kernel density estimates (KDEs), with 95% CIs calculated using 100 bootstrap samples with replacement. KDEs were calculated with the reference smoothing parameter *h‐ref* which is suited to small sample sizes and reduced over‐smoothing of data (Borger et al., 2006; Fieberg & Kochanny, 2005; Laver & Kelly, 2008), in the *R* package *adehabitatHR* (Calenge, 2012). We investigated the effect of sex, year of release, distance to settlement (*d*), time to settlement (*t*), and an interaction between year of release and sex on the mean home range size of martens using a Gaussian linear model. Range size was log‐transformed to normalize the distribution of residuals. Model selection was undertaken using backward stepwise elimination as above.

### 2.4 Habitat preference

We investigated habitat preference in a hierarchical framework, following definitions by Johnson (1980), which provide that selection on a landscape scale by individuals has already occurred during the establishment of a home range (first‐order selection). The regional habitat composition therefore already determines a degree of habitat selection of individuals; however, we can investigate selection at different spatial scales (McLoughlin et al., 2002; Rettie & Messier, 2000; Virgos, Zalewski, Rosalino, & Mergey, 2012; Johnson, 1980). We thus examined the second‐order habitat selection (i.e., habitat within the home range) by investigating the broad habitat types used by individuals in their home range, similar to approaches by Rondinini and Boitani (2002). We then focused on forested areas and examined third‐order selection (i.e., the usage of components within the home range) to investigate whether there was any selection for particular woodland types. In both instances, we asked whether certain habitat types were used disproportionately to their availability.

Preferences for broad habitat types (second‐order habitat selection) and then for forest types (third‐order habitat selection) were investigated separately. Geo‐referenced land‐use data were obtained from the CORINE Land Cover (CLC) 2012 database (scale 1:100,000; created in 2011–2012, released in 2016). Land‐use classifications were grouped into three biologically relevant classes: agricultural land, forest, and grassland (Appendix S1). Forest‐type data were acquired from the National Forest Inventory (NFI) 2016 database (created and released in 2016; Forestry Commission 2016). Forest‐type classifications were condensed into five major groups: broadleaf, conifer, felled, open areas, and young or sparse woodland (Appendix S1).

The habitat preferences of all pine martens (for both broad land‐use and forest type) during the postrelease “settlement” period, up to 100 days postrelease, were assessed using a use‐availability design, where preference is the ratio of used to available habitat (Aebischer, Robertson, & Kenward, 1993; Warton & Aarts, 2013). We compared the habitat types and characteristics of “used” locations with “available” habitat at randomly sampled locations. This approach therefore considered the landscape composition but not the landscape structure, that is, patchiness of habitat types. Available habitat was sampled randomly from a uniformly sized area around the home range centroid of each marten. The radius of this area (7.15 km) was defined by calculating the mean maximum Euclidean distance that each marten with an adequate fix number was located from their home range centroid. To ensure thorough representation of “available” habitat, each “used” location had five corresponding “available” locations. This unequal ratio was then accounted for by weighting locations within subsequent models so that five “available” points were equivalent to one “used” point. Both “used” and “available” points were overlaid on habitat spatial polygons, and the underlying habitat‐type data were extracted. The total area of “available” habitat assessed in this study was 1,404 km^2^. The total area assessed comprised urban areas (~1%), wetland/bog (11%), forest (12%), grassland (30%), and agricultural land (46%). Fewer than 10 marten locations were found in wetland/bog and urban areas, as a result these broad habitat types were excluded from analyses. For analysis of forest‐type preference, available locations were only generated within NFI forest polygons to ensure complete representation of available forested habitat. For individuals situated close to the coast, areas were clipped to avoid selection of the marine environment and intertidal zones (< 500 m of the low water mark). All habitat use data were processed using the *R* package *sp* (Pebesma & Bivand, 2012).

We fitted generalized estimating equations (GEEs) in a general linear model (GLM) framework to investigate the habitat preference of martens in different release years and between sexes. GEEs enhance GLMs by accounting for the spatial and temporal autocorrelation within locations recorded for individuals. The assumption of independence, made in a GLM, is replaced with a correlation structure that groups individuals, allowing for correlation within, but not between, individuals. GEE‐GLMs use the empirical standard error in analysis, which is more robust to misspecification of correlation structure and nonindependence of data points, an inherent feature of telemetry data (Booth, Embling, Gordon, Calderan, & Hammond, 2013; Zuur, Ieno, Walker, Saveliev, & Smith, 2009). Incorporating these correlation structures makes it possible to generate a population mean response rather than making inferences about single individuals (Braaker et al., 2014; Zuur et al., 2009). GEE‐GLMs with a binomial error distribution and logit link function were used to model the habitat preference of pine martens. The response variable was binary: used versus*.* available. Habitat type and its interaction with both release year and sex were factor variables. The weight of the point (used = 1, available = 0.2) was also specified. Release year, either 2015 or 2016, was included to test for variance arising from (1) differences in release sites between years and (2) the presence of conspecifics in the second year of releases. Animals released in 2015 (year 1) were not monitored in 2016, and therefore, each year contains a different set of newly released individuals. Individual martens were defined as clusters, and the correlation structure was assumed to be independent, that is, correlation structure was expected among locations from the same individual but not between individuals (Braaker et al., 2014; Fieberg & Kochanny, 2005; Pirotta, Matthiopoulos, Mackenzie, Scott‐Hayward, & Rendell, 2011).

Models contained fixed effects of sex, habitat type, and year. We included all main effects and the two‐way interactions between sex and habitat type, and year and habitat type. We used backward‐step selection using GEE‐GLM *p*‐values to obtain the minimum adequate model. Models were assessed using Wald's tests (GEE‐GLM ANOVA function in *geepack*) to ensure that all retained variables had a *p*‐value < .05 (Ventura, Matthiopoulos, & Jeglinski, 2019). Based on the significance of an interaction term, data from each year or sex were then modeled separately to identify the differences in preference within each group. Parametric bootstrapping 1,000 times using GEE‐based uncertainty parameters was implemented to calculate 95% CIs around the population mean (Pirotta et al., 2011; Russell et al., 2015). All models were fitted using the *geeglm* function in the *geepack* package (Halekoh, 2006) in *R* version 3.5.1.

### 2.5 Ethical statement

The study was approved by the University of Exeter Animal Welfare and Ethical Review Board and was conducted under licenses from Scottish Natural Heritage and Natural Resources Wales and from The Home Office under the Animals (Scientific Procedures) Act.

## 3 RESULTS

In 2015 and 2016, 39 pine martens (10 M and 10F in 2015 and 10 M and 9F in 2016) were translocated from Scotland and released into mid‐Wales. Ten individuals were completely excluded from subsequent analyses due to an inadequate number of fixes (<6) within 100 days. This was a result of either shedding the radio collar in the release pen (*n = *1), mortality (*n = *3; two individuals died after 13 days due to infection and one individual was thought to have been killed by a predator after 16 days), or inability to relocate animals for a long period of time immediately after release (*n = *6; although 4 of these were subsequently relocated and identified 27–230 days postrelease). Within the first 100 days postrelease, the mean number of fixes recorded for the 29 (6M and 7F in 2015 and 9M and 7F in 2016) successfully tracked individuals was 35 (*SD* = 20 fixes; range = 7–84; Appendix S3).

**FIGURE 3:**
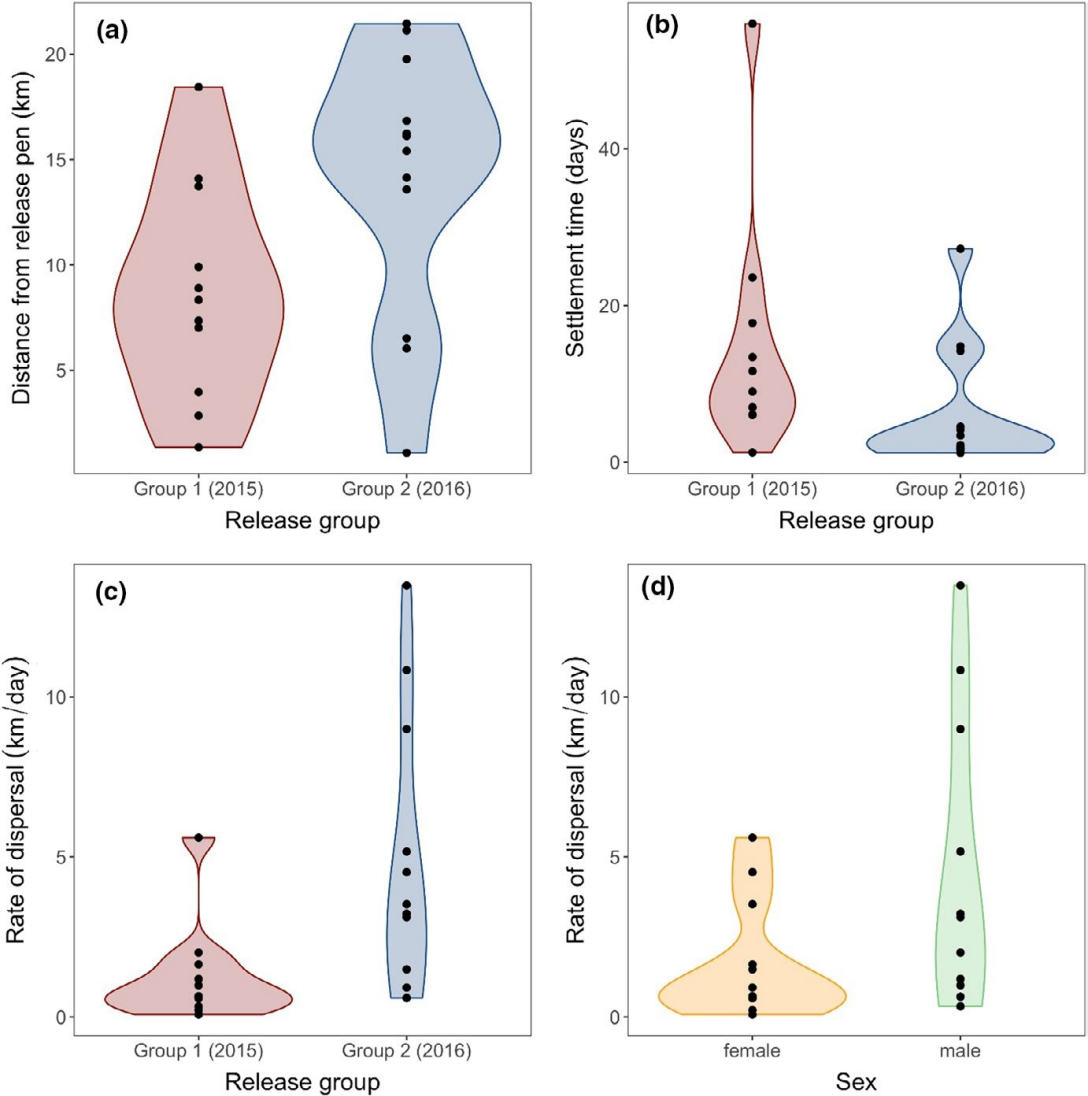
Summaries of postrelease movement of translocated pine martens away from release pens over 100 days after release. (a) Distance (*d*) from release pen (km) at which pine martens switched from the “exploration” phase and entered the “settlement” phase during which they established stable home ranges. (b) Time (*t*) since release (days) at which pine martens switched from the “exploration” phase and entered the “settlement” phase during which they established stable home ranges. (c) Rate (*r*, in km/day) that pine martens dispersed from their release pen before entering the settlement phase. (d) Rate (*r*, in km/day) that female and male pine martens dispersed from their release pens. The first release group (2015) is shown in red, and the second group (2016) is shown in blue. Females are shown in orange, and males are shown in green. Raw data are shown in black

We identified two clear stages of postrelease movement by translocated pine martens within the first 100 days postrelease, of “exploration” followed by “settlement.” For 23 of the 29 pine martens, a segmented linear model with two stages characterized marten movements postrelease better (lower AICc score) than a simple linear regression (Figure 2). The distance (*d*) and time (*t*) taken to settlement differed significantly between the two release years (Figure 3a and b), while the rate of exploration (*r*) varied both with year of release and pine marten sex (Figure 3c and d). The minimum adequate model for settlement time identified an effect of year of release on settlement time (*t*) ( $\chi_{2,1}^{2}$ = 3.83, *p* = .05). Pine martens released in the second year took significantly less time to settle than those released in the first (Figure 3b). Settlement occurred at a mean of 14.5 days (*SE* = 3.9 days) in the first year, compared to 6.6 days (*SE* = 1.8 days) in the second year. The longest time taken to settle by an individual was 56 days. There was no difference between the sexes ( $\chi_{2,1}^{2}$ = 0.078, *p* = .78). The minimum adequate model for settlement distance (*d*) showed that year of release significantly affected settlement distance ( $\chi_{2,1}^{2}$ = −161.48, *p* = .03). Pine martens released in the first year settled closer to their point of release than those in the second year (Figure 3a). Animals in the first year settled a mean of 8.7 km (*SE* = 1.8 km) away from the release site, whereas animals in the second year traveled a mean of 14.0 km (*SE* = 1.7 km; Figure 3a). The maximum distance at which the tracked martens settled within 100 days was 21.5 km, and the minimum was 1.1 km. There was no difference in settlement distance between male and female martens ( $\chi_{2,1}^{2}$ = −115.01, *p* = .074). Of the 6 individuals that were not located immediately after release, 4 were later found 1.0–103.0 km from their release locations. The minimum adequate model for exploration rate (*r*) included effects of year of release ( $\chi_{2,1}^{2}$ = −10.92, *p* = .001) and sex ( $\chi_{2,1}^{2}$ = −5.22, *p* = .026). When averaged over sex, animals released in the second year dispersed from their point of release at a greater rate than those released in the first year (Figure 3c). Year one animals traveled at a rate of 0.75 km/day (*SE* = 0.23 km/day) compared to year two animals at a rate of 3.00 km/day (*SE* = 0.89 km/day). When averaged over years, males also showed a significantly greater rate of dispersal than females (Figure 3d). Females traveled at a mean rate of 0.93 km/day (*SE* = 2.76 km/day), whereas males traveled at 2.42 km/day (*SE* = 0.75 km/day) on average.

**FIGURE 4:**
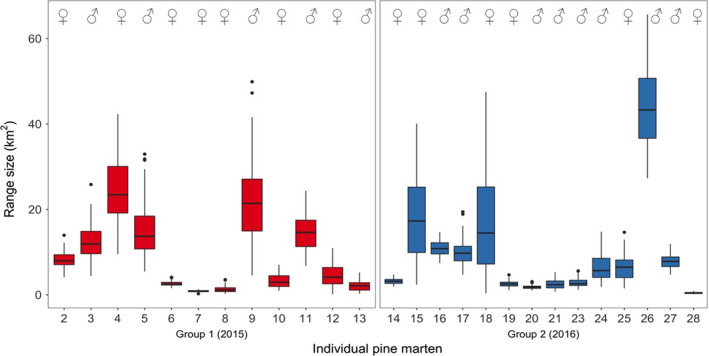
Home range sizes of translocated pine martens calculated using locations recorded from the time of settlement up until 100 days postrelease. Tops and bottoms of the bars represent the 75th and 25th percentiles of the data, the black lines are the medians, and the whiskers extend from their respective hinge to the smallest or largest value, no further than 1.5 times that of the interquartile range. Points outside this range are outliers. The first release group (2015) is shown in red, and the second release group (2016) is in blue. Individual pine marten numbers correspond to animals in Figure 1. Martens 1, 22, and 29 were excluded from range calculations due to an inadequate number of locations collected postsettlement

The mean home range size of martens in the settlement phase (i.e., from the breakpoint up to 100 days) was 9.5 km^2^ (*SD* = 10.6 km^2^, range = 0.2–65.6 km^2^, *n* = 26; Figure 4). Variation in range size was not significantly affected by sex, year of release, the interaction between sex and year of release, settlement time, or settlement distance.

**FIGURE 5:**
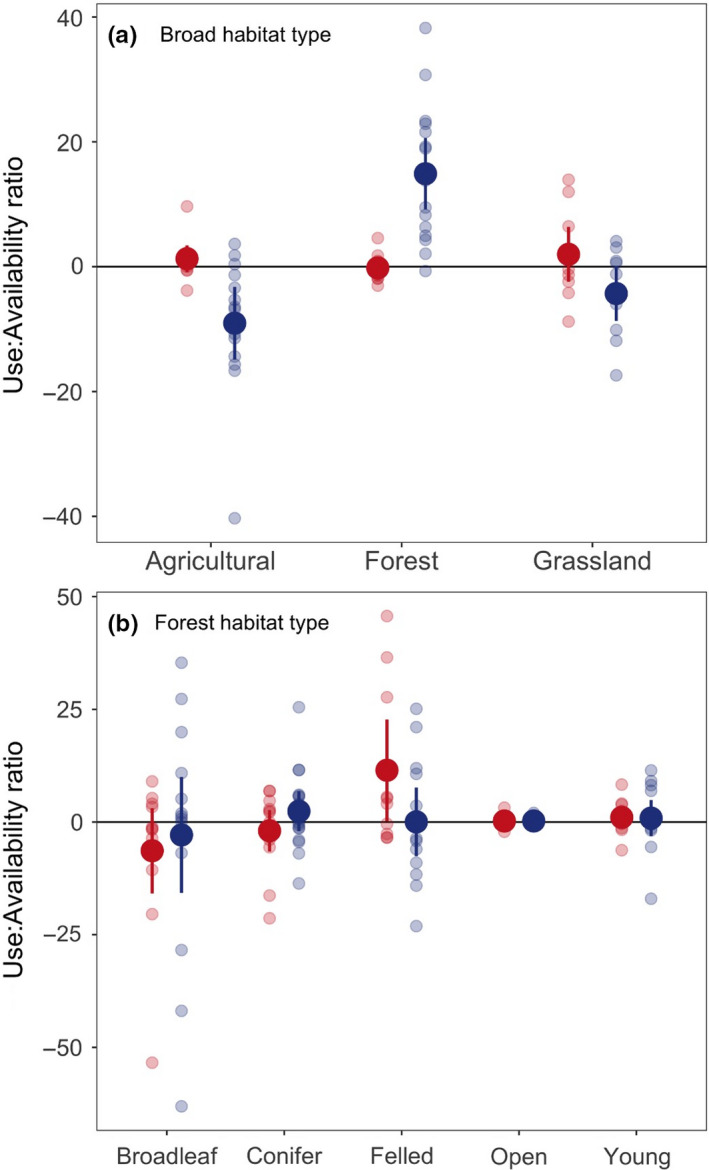
Habitat preferences of translocated pine martens released in year one (2015; red) and two (2016; blue)**.** Top plot shows broad‐scale habitat preferences, and bottom plot shows forest habitat preferences. Plots show the ratio of use to availability of habitat types plotted on the scale of the response. Mean values and 95% confidence intervals are shown in bold. Raw data for each marten are shown by small points. A value of 0 indicates use of a habitat in equal proportion to its availability. Positive values indicate preferential use of a habitat type in relation to its availability. Negative values indicate lower use of a habitat than expected in relation to its availability

The preference of martens for broad habitat types after settlement and up to 100 days since release differed significantly between release years (GEE‐GLM; $\chi_{3}^{2}$ = 55.2, *p* < .001). When broad habitat‐type preference was assessed separately for each year, pine martens did not display a strong habitat preference in year one, but in the second year martens preferred forest habitats and avoided agricultural areas and grassland (GEE‐GLM; $\chi_{2}^{2}$ = 76.6, *p* < .001; Figure 5a). Marten preferences for forest type also differed between years (GEE‐GLM; $\chi_{5}^{2}$ = 17.15, *p* = .004, Figure 5b). When each year group was assessed separately, martens showed strong preference for felled areas in year one (GEE‐GLM; $\chi_{4}^{2}$ = 28.9, *p < *.001; Figure 5b), while in the second year, martens did not show preference for any forest types.

## 4 DISCUSSION

Postrelease movement of translocated martens followed distinct patterns, and the presence of previously released conspecifics altered the duration and extent of dispersal by individuals in a subsequent release. This in turn influenced home range location and resulting habitat use. Animals released in phases should thus not be expected to follow identical postrelease behavioral patterns, but instead are influenced by the presence and location of conspecifics.

We observed a clear, two‐phase, postrelease movement pattern undertaken by pine martens translocated from their core range in Scotland to mid‐Wales. This pattern comprised exploration followed by settlement and was likely a result of initial searching of the new environment for denning and foraging habitat (Moehrenschlager & Macdonald, 2003; Sjoasen, 1997; Slough, 1989; Stamps, 2001). A switch to settlement suggests identification of adequate habitat in which to establish a territory. Postrelease movement strategies differed between subsequent years of release, with animals traveling further and faster before settling in year two. Here, the main period of exploration predominantly occurred within the first two weeks postrelease. Intensive tracking of animals within this initial time period is therefore clearly desirable to avoid loss of contact with dispersing animals. Preferences for broad‐scale habitat and forest‐type also differed between years. It is likely that conspecific density, habitat quality, and landscape structure are major factors influencing these differences.

The initial retention of translocated individuals closer to their release sites is central to the long‐term viability and establishment of a new population (Yott et al., 2011). Although they differed between years, in Wales the mean distances of pine marten dispersal prior to settlement (8 km for 2015 releases and 14.0 km for 2016 releases) were comparable to those recorded for *Martes americana* translocations over similar time periods (0.4–75.3 km within 4–161 days; (Davis, 1983), 0.4–45.7 km within 1–64 days; (Woodford et al., 2013)). Year 1 individuals established territories near to their release sites (Figure 1). Although consisting of large forestry blocks, these release sites are surrounded by pasture, moorland, and farmland. Such areas were selected for marten release as they provided a diverse structural environment required for denning, combined with fields and edge‐habitat in which to forage, resulting in the use of habitat proportional to its availability. Recent studies provide supporting evidence that pine martens are less habitat‐specific than previously thought (Manzo et al., 2018), suggesting that a combination of habitat types is required. This landscape complementation (Dunning, Danielson, & Pulliam, 1992) enables individuals to utilize different habitat types for different functions or key resources. For example, within large compartments of commercially managed forestry, tree thinning and felling are common. The felled woodland, favored by animals released in the first year (Figure 5b), often comprises wind‐thrown trees or large areas of debris and offers structural complexity utilized by martens for denning and foraging (Caryl et al., 2012; Clevenger, 1994; Lombardini et al., 2015). Growth of new vegetation as a result of felling has been shown to increase diversity and biomass of rodent species, the primary food source of martens (Caryl et al., 2012; Sidorovich, Sidorovich, & Krasko, 2010). In newly felled areas, martens have been found to respond to this through increased consumption not just of field voles *Microtus agrestis* but also of bank voles *Myodes glareolus* and wood mice *Apodemus sylvaticus* (Sidorovich et al., 2010; Steventon & Major, 1982). The preferential use of these areas by martens in the first year of the releases may therefore be a result of high prey abundance in close proximity to denning sites.

As marten density in the release area increased as a result of territorial establishment by year one individuals, animals released under the same protocols and conditions in year two likely dispersed further in response to territory or site saturation and competition for resources (Woodford et al., 2013; Yott et al., 2011). A study on released otters similarly found that the movement (i.e., exploration) distance of individuals released into unoccupied areas was much lower than those released into areas containing conspecifics (Sjoasen, 1997). Density‐dependent dispersal (Massaro, Chick, Kennedy, & Whitsed, 2018) is therefore a likely driver of greater settlement distance in second‐year animals. However, these second‐year animals did settle faster than those released in the first year (Figure 3b), possibly spending less time searching for appropriate habitat near to release sites and dispersing immediately out of the large forest blocks into empty territories. These individuals quickly settled in smaller forest fragments on the periphery of the core population (Figure 1), suggestive of saturation around the release sites. The mosaic structures made up of noncommercial woodland, scattered within and around areas dominated by farmland, explain the broad‐scale preferential use of forested habitat but lack of selectivity of forest type. The likely use of mosaic habitats, particularly in year two, may have been driven by landscape structure, that is, not only habitat composition but the way in which habitat types are distributed across an area. Animals may cover larger areas comprising multiple small patches of preferred habitat, interspersed with low‐quality habitat. This strategy, known as landscape supplementation (Dunning et al., 1992), enables the persistence of individuals where large, contiguous habitat is not available and can also facilitate dispersal.

Movement of some individuals was unpredictable and, in both years, a small number of martens (six individuals in total; 15% of 39 animals) were lost after release. Four of these individuals were found again after a long period of absence, some having traveled exceptionally long distances (e.g., one individual was relocated 103 km away from its release site 172 days postrelease). When a population is in flux, processes such as habitat preference and range size may be less predictable, demonstrated here as animals try to re‐establish themselves, with a lack of mutually exclusive ranges in individuals released in year one (Figure 1). High numbers of these long‐distance dispersers may be detrimental to the viability of the translocated populations. With the next nearest established population of martens located in Kielder forest, over 300 km away, the likelihood of new individuals arriving in the area and compensating for loss of highly dispersive translocated individuals is negligible (Mihoub et al., 2011). This long‐distance dispersal has been observed in slightly higher proportions in other translocation studies of marten species (26%; Davis, 1983, 30%; Slough, 1989) and is often indicative of local territorial saturation (Yott et al., 2011). Here, the driving forces behind long‐distance dispersal remain unclear, although it has been suggested that individual personality and stress levels may be influential (Clobert, Galliard, Cote, Meylan, & Massot, 2009). The drivers of range size variation were also unclear and could not be attributed to sex, year of release or any postrelease metrics. The home range sizes estimated for settled martens were, however, similar to those previously recorded for martens in source locations (5.6–23.6 km^2^; Caryl, 2008). On visual inspection, these range sizes of martens do show overall differences, with ranges being more defined and apparent in year two individuals who show distinct territorial formation akin to those typical of established populations (Balharry, 1993; Powell, 1979; Figure 1). This may potentially be a result of stronger territorial distinction by established individuals in their second year, when sex‐based differences in ranging become more apparent prior to mating and offspring being born in following years (Erlinge & Sandell, 1986; Powell, 1979; Sjoasen, 1997; Slough, 1989; Tolhurst et al., 2015; Yott et al., 2011). However, the density of martens in the recipient region thus far, approximately 0.03 martens/km^2^, is substantially lower than elsewhere across the species range in Ireland (1.25–4.42 martens/km^2^, Sheehy, O’Meara, O’Reilly, Smart, & Lawton, 2014) or Scotland (0.16–0.28, Balharry, 1993; 0.32–0.46, Halliwell, 1997; 0.28–2.0, Caryl, 2012). Since martens in this study were sourced from various Scottish locations, conspecific density, and therefore home range size, was likely variable and may influence initial ranging behavior. With time, it is expected that marten range size at release sites will stabilize in accordance with the local density of individuals.

The difference in postrelease strategies by year one and year two animals in this study suggests that the role of conspecifics, particularly established residents, can influence posttranslocation movement by released animals. In translocation projects, release of animals is frequently performed in phases due to logistical constraints (Richardson & Ewen, 2016). There is often an assumption that individuals in initial and subsequent releases will behave in a comparable manner (Richardson et al., 2016). However, as shown in this study, the presence or absence of an established population can result in different dispersal strategies (Richardson & Ewen, 2016). The response of released animals to conspecific presence and density should thus be central to reintroduction planning (Richardson & Ewen, 2016). Reinforcement of social or colonial species can exploit conspecific attraction to aid the success of projects, either translocating animals in family units or releasing individuals into pre‐established colonies (Ward & Schlossberg, 2004). The presence of other individuals can indicate suitability of habitat as well as mate availability, having an anchoring effect on subsequently released animals (Ward & Schlossberg, 2004). Even in mammals that are not obviously social or colonial, such as the pine marten, social information is still important in dispersal decisions.

Translocation and release of animals require consideration of the social structure and demographic processes driving movement and ranging behavior. In a translocated population, however, this social structure is initially undefined and can result in unpredictable responses to conspecifics and increased dispersal or mortality, particularly if neighbors are unfamiliar (Richardson & Ewen, 2016; Shier & Swaisgood, 2011). Conspecific attraction might, however, improve the establishment of a release‐site population and can be achieved through i) translocation of large numbers of individuals, such as in year one of this study, ii) translocation of neighboring individuals from source sites (Shier & Swaisgood, 2011; Ydenberg et al., 1988), although this may result in a higher level of relatedness among individuals, or iii) translocation of individuals into pre‐established, low‐density populations, such as in year two of this study (Richardson & Ewen, 2016).

## CONFLICT OF INTEREST

The authors declare no conflicts of interest.

## AUTHOR CONTRIBUTIONS

**Catherine M. McNicol:** Conceptualization (equal); Data curation (equal); Formal analysis (lead); Investigation (lead); Methodology (lead); Visualization (equal); Writing‐original draft (lead). **David Bavin:** Data curation; Investigation (equal); Methodology (equal); Writing‐review & editing (supporting). **Stuart Bearhop:** Conceptualization; Supervision (equal); Writing‐review & editing (equal). **Josie Bridges:** Investigation (equal). **Elizabeth Croose:** Investigation (equal); Methodology (equal); Writing‐review & editing (equal). **Robin Gill:** Conceptualization (equal); Supervision (equal); Writing‐review & editing (supporting). **Cecily E. D. Goodwin:** Formal analysis (supporting); Methodology (supporting); Software (supporting). **John Lewis:** Investigation; Methodology (equal). **Jenny Macpherson:** Conceptualization (equal); Funding acquisition (equal); Investigation; Methodology (equal); Project administration (equal); Supervision (equal); Writing‐review & editing (equal). **Daniel Padfield:** Methodology (equal); Resources (equal); Software (equal); Writing‐review & editing (equal). **Henry Schofield:** Funding acquisition (equal); Investigation; Methodology (equal); Project administration (equal); Supervision (equal); Writing‐review & editing (equal). **Matthew J. Silk:** Formal analysis (equal); Software (equal). **Alexandra J. Tomlinson:** Investigation; Methodology (equal); Project administration (equal). **Robbie A. McDonald:** Conceptualization (equal); Funding acquisition (equal); Investigation; Project administration (equal); Supervision (equal); Writing‐original draft (supporting); Writing‐review & editing (equal).

5

## Supporting information

Supplementary Material

## Data Availability

Data available from the Dryad Digital Repository: https://doi.org/10.5061/dryad.3tx95x6c4 (McNicol et al., 2020).

## References

[ece36265-bib-0001] Aebischer, N. J. , Robertson, P. A. , & Kenward, R. E. (1993). Compositional analysis of habitat use from animal radio‐tracking data. Ecology, 74, 1313–1325.

[ece36265-bib-0002] Armstrong, D. P. , Mcarthur, N. , Govella, S. , Morgan, K. , Johnston, R. , Gorman, N. , … Richard, Y. (2013). Using radio‐tracking data to predict post‐release establishment in reintroductions to habitat fragments. Biological Conservation, 168, 152–160. 10.1016/j.biocon.2013.09.028

[ece36265-bib-0003] Armstrong, D. P. , & Seddon, P. J. (2008). Directions in reintroduction biology. Trends in Ecology & Evolution, 23, 20–25. 10.1016/j.tree.2007.10.003 18160175

[ece36265-bib-0004] Balestrieri, A. , Remonti, L. , Ruiz‐Gonzalez, A. , Gomez‐Moliner, B. J. , Vergara, M. , & Prigioni, C. (2010). Range expansion of the pine marten (*Martes martes*) in an agricultural landscape matrix (NW Italy). Mammalian Biology, 75, 412–419. 10.1016/j.mambio.2009.05.003

[ece36265-bib-0005] Balharry, D. (1993). Factors affecting the distribution and population density of pine martens *Martes martes* in Scotland. PhD Thesis, University of Aberdeen.

[ece36265-bib-0006] Bartolommei, P. , Manzo, E. , & Cozzolino, R. (2016). Seasonal spatial behaviour of pine marten *Martes martes *in a deciduous oak forest of central Italy. Mammal Research, 61, 319–326.

[ece36265-bib-0007] Birks, J. D. S. , & Kitchener, A. C. (1990). The Distribution and Status of the Polecat *Mustela putorius* in Britain in the 1990s. Vincent Wildlife Trust.

[ece36265-bib-0008] Birks, J. D. S. , & Messenger, J. E. (2010). Evidence of Pine Martens in England and Wales 1996–2007. Vincent Wildlife Trust.

[ece36265-bib-0009] Booth, C. G. , Embling, C. , Gordon, J. , Calderan, S. V. , & Hammond, P. S. (2013). Habitat preferences and distribution of the harbour porpoise *Phocoena phocoena* west of Scotland. Marine Ecology Progress Series, 478, 273–285.

[ece36265-bib-0010] Borger, L. , Franconi, N. , Ferretti, F. , Meschi, F. , Michele, G. D. , Gantz, A. , & Coulson, T. (2006). An integrated approach to identify spatiotemporal and individual‐level determinants of animal home range size. American Midland Naturalist, 168, 471–485. 10.1086/507883 17004219

[ece36265-bib-0011] Braaker, S. , Moretti, M. , Boesch, R. , Ghazoul, J. , Obrist, M. K. , & Bontadina, F. (2014). Assessing habitat connectivity for ground‐dwelling animals in an urban environment. Ecological Applications, 24, 1583–1595. 29210224 10.1890/13-1088.1

[ece36265-bib-0012] Bright, P. W. , & Smithson, T. J. (1997). Species Recovery Programme for the pine marten in England: 1995–96).English Nature.

[ece36265-bib-0013] Broquet, T. , Johnson, C. A. , Petit, E. , Thompson, I. , Burel, F. , & Fryxell, J. M. (2006). Dispersal and genetic structure in the American marten, *Martes americana* . Molecular Ecology, 15, 1689–1697. 10.1111/j.1365-294X.2006.02878.x 16629821

[ece36265-bib-0014] Burnham, K. P. , & Anderson, D. R. (2004). Multimodel inference: Understanding AIC and BIC in model selection. Sociological Methods & Research, 33, 261–304. 10.1177/0049124104268644

[ece36265-bib-0015] Calenge, A. C. 2012 Package ‘ adehabitatHR’.

[ece36265-bib-0016] Caryl, F. M. (2008). Pine marten diet and habitat use within a managed coniferous forest. PhD Thesis, University of Stirling.

[ece36265-bib-0017] Caryl, F. M. , Quine, C. P. , & Park, K. J. (2012). Martens in the matrix: The importance of nonforested habitats for forest carnivores in fragmented landscapes. Journal of Mammalogy, 93, 464–474.

[ece36265-bib-0018] Clevenger, A. P. (1994). Habitat characteristics of Eurasian pine martens *Martes martes* in an insular Mediterranean environment. Ecography, 17, 257–263.

[ece36265-bib-0019] Clobert, J. , Le Galliard, J. F. , Cote, J. , Meylan, S. , & Massot, M. (2009). Informed dispersal, heterogeneity in animal dispersal syndromes and the dynamics of spatially structured populations. Ecology Letters, 12, 197–209. 19170731 10.1111/j.1461-0248.2008.01267.x

[ece36265-bib-0020] Davis, M. H. (1983). Post‐release movements of introduced marten. Journal of Wildlife Management, 47, 59–66.

[ece36265-bib-0021] Dunning, J. B. , Danielson, B. J. , & Pulliam, H. R. (1992). Ecological processes that affect populations in complex landscapes. Oikos, 65, 169–175.

[ece36265-bib-0022] Erlinge, S. , & Sandell, M. (1986). Seasonal changes in the social organization of male stoats, Mustela erminea: An effect of shifts between two decisive resources. Oikos, 47, 57–62.

[ece36265-bib-0023] Fieberg, J. , & Kochanny, C. (2005). Quantifying home‐range overlap: The importance of the utilization distribution. Journal of Wildlife Management, 69, 1346–1359.

[ece36265-bib-0024] Fretwell, S. D. , & Lucas, H. L. (1962). On territorial behaviour and other factors influencing habitat distribution in birds: Theoretical development. Acta Biotheoretica, 19, 16–36.

[ece36265-bib-0025] Halekoh, U. (2006). The R Package geepack for Generalized Estimating Equations. Journal of Statistical Software, 15, 9–10.

[ece36265-bib-0026] Halliwell, E.C. (1997). The ecology of red squirrels in Scotland in relation to pine marten predation. PhD thesis. University of Aberdeen, Aberdeen, UK.

[ece36265-bib-0027] Hansson, L. (1978). Small mammal abundance in relation to environmental variables in three Swedish forest phases. Studia Forestalia Suedica, 147, 1–40.

[ece36265-bib-0028] Hayward, M. W. , O’Brien, J. , Hofmeyr, M. , Kerley, G. I. H. , Adendorff, J. , Moolman, L. C. , … Slater, R. (2007). The reintroduction of large carnivores to the Eastern Cape, South Africa: An assessment. Oryx, 71, 205–214.

[ece36265-bib-0029] IUCN (2013). Guidelines for reintroductions and other conservation translocations. Version 1.0. Gland, Switzerland: IUCN Species Survival Commission.

[ece36265-bib-0030] Jefferies, D. J. , Wayre, P. , & Jessop, R. M. (1986). Reinforcing the native Otter *Lutra lutra* population in East Anglia: An analysis of the behaviour and range development of the first release group. Mammal Review, 16, 65–79.

[ece36265-bib-0031] Johnson, D. H. (1980). The comparison of usage and availability measurements for evaluating resource preference. Ecology, 61, 65–71.

[ece36265-bib-0032] Jordan, N. R. , Messenger, J. , Turner, P. , Croose, E. , Birks, J. , & Reilly, C. O. (2012). Molecular comparison of historical and contemporary pine marten (*Martes martes*) populations in the British Isles: Evidence of differing origins and fates, and implications for conservation management. Conservation Genetics, 13, 1195–1212.

[ece36265-bib-0033] Laver, P. N. , & Kelly, M. J. (2008). A critical review of home range studies. Journal of Wildlife Management, 72, 290–298.

[ece36265-bib-0034] Lenth, R. , Singmann, H. , Love, J. , Buerkner, P. , & Herve, M. (2019). Package “emmeans”.

[ece36265-bib-0035] Letty, J. , Marchandeau, S. , & Aubineau, J. (2007). Problems encountered by individuals in animal translocations: Lessons from field studies. Ecoscience, 14, 259–271.

[ece36265-bib-0036] Lombardini, M. , Cinerari, C. E. , Murru, M. , Vidus Rosin, A. , Mazzoleni, L. , & Meriggi, A. (2015). Habitat requirements of Eurasian pine marten *Martes martes* in a Mediterranean environment. Mammal Research, 60, 97–105.

[ece36265-bib-0037] MacPherson, J. (2014). Feasibility assessment for reinforcing pine marten numbers in England and Wales. Ledbury, UK: Vincent Wildlife Trust.

[ece36265-bib-0038] Manzo, E. , Bartolommei, P. , Giuliani, A. , Gentile, G. , Dessi‐Fulgheri, F. , & Cozzolino, R. (2018). Habitat selection of European pine marten in Central Italy: From a tree dependent to a generalist species. Mammal Research, 63, 357–367.

[ece36265-bib-0039] Manzo, E. , Bartolommei, P. , Rowcliffe, J. M. , & Cozzolino, R. (2012). Estimation of population density of European pine marten in central Italy using camera trapping. Acta Theriologica, 57, 165–172.

[ece36265-bib-0040] Marschner, I. , Donoghoe, M. W. , & Donoghoe, M. M. W. (2018). Package “glm2”.

[ece36265-bib-0041] Massaro, M. , Chick, A. , Kennedy, E. S. , & Whitsed, R. (2018). Post‐reintroduction distribution and habitat preferences of a spatially limited island bird species. Animal Conservation, 21, 54–64.

[ece36265-bib-0042] Mccann, N. P. , Zollner, P. A. , & Gilbert, J. H. (2017). Temporal scaling in analysis of animal activity. Ecography, 40, 1436–1444.

[ece36265-bib-0043] McLoughlin, P. D. , Case, R. L. , Gau, R. J. , Cluff, D. H. , Mulders, R. , & Messier, F. (2002). Hierarchical habitat selection by barren‐ground grizzly bears in the central Canadian Arctic. Oecologia, 132, 102–108. 10.1007/s00442-002-0941-5 28547280

[ece36265-bib-0044] McNicol, C. M. , Bavin, D. , Bearhop, S. , Bridges, J. , Croose, E. , Gill, R. , … McDonald, R. A. (2020). Data from: Post‐release movement and habitat selection of translocated pine martens *Martes martes* . Dryad Digital Repository, 10.5061/dryad.3tx95x6c4 PMC7297779 32551086

[ece36265-bib-0045] Mihoub, J. B. , Robert, A. , Le Gouar, P. , & Sarrazin, F. (2011). Post‐release dispersal in animal translocations: Social attraction and the “Vacuum Effect”. PLoS ONE, 6, e27453. 22194784 10.1371/journal.pone.0027453 PMC3237406

[ece36265-bib-0046] Moehrenschlager, A. , & Macdonald, D. W. (2003). Movement and survival parameters of translocated and resident swift foxes *Vulpes velox* . Animal Conservation, 6, 199–206. 10.1017/S1367943003251

[ece36265-bib-0047] Moll, R. J. , Kilshaw, K. , Montgomery, R. A. , Abade, L. , Campbell, R. D. , Harrington, L. A. , … Macdonald, D. W. (2016). Clarifying habitat niche width using broad‐scale, hierarchical occupancy models: A case study with a recovering mesocarnivore. Journal of Zoology, 300, 177–185. 10.1111/jzo.12369

[ece36265-bib-0048] Muggeo, M. V. M. R. (2017). Package ‘ segmented’.

[ece36265-bib-0049] Pebesma, E. , & Bivand, R. (2012). Package ‘sp’.

[ece36265-bib-0050] Pereboom, V. , Mergey, M. , Villerette, N. , Helder, R. , Gerard, J.‐F. , & Lodé, T. (2008). Movement patterns, habitat selection, and corridor use of a typical woodland‐dweller species, the European pine marten (*Martes martes*), in fragmented landscape. Canadian Journal of Zoology, 86, 983–991. 10.1139/Z08-076

[ece36265-bib-0051] Pirotta, E. , Matthiopoulos, J. , Mackenzie, M. , Scott‐Hayward, L. , & Rendell, L. (2011). Modelling sperm whale habitat preference: A novel approach combining transect and follow data. Marine Ecology Progress Series, 436, 257–272. 10.3354/meps09236

[ece36265-bib-0052] Powell, R. A. (1979). Mustelid spacing patterns: Variations on a theme by *Mustela* . Zeitschrift Für Tierpsychologie, 50, 153–165. 10.1111/j.1439-0310.1979.tb01023.x

[ece36265-bib-0053] R Core Team (2018). R: A language and environment for statistical computing. Vienna, Austria: R Foundation for Statistical Computing https://www.R‐project.org/

[ece36265-bib-0054] Rettie, W. J. , & Messier, F. (2000). Hierarchical habitat selection by woodland caribou: Its relationship to limiting factors. Ecography, 23, 466–478.

[ece36265-bib-0055] Richardson, K. M. , & Ewen, J. G. (2016). Habitat selection in a reintroduced population: Social effects differ between natal and post‐release dispersal. Animal Conservation, 19, 413–421. 10.1111/acv.12257

[ece36265-bib-0056] Robertson, C. P. J. , & Harris, S. (1995). The behaviour after release of captive‐reared fox cubs. Animal Welfare, 4, 295–306.

[ece36265-bib-0057] Rondinini, C. , & Boitani, L. (2002). Habitat use by beech martens in a fragmented landscape. Ecography, 25, 257–264.

[ece36265-bib-0058] Russell, D. J. F. , Mcclintock, B. T. , Matthiopoulos, J. , Thompson, P. M. , Thompson, D. , Hammond, P. S. , … Mcconnell, B. J. (2015). Intrinsic and extrinsic drivers of activity budgets in sympatric grey and harbour seals. Oikos, 124, 1462–1472. 10.1111/oik.01810

[ece36265-bib-0059] Sainsbury, K. A. , Shore, R. F. , Schofield, H. , Croose, E. , Campbell, R. D. , & McDonald, R. A. (2019). Recent history, current status, conservation and management of native mammalian carnivore species in Great Britain. Mammal Review, 49, 171–188. 10.1111/mam.12150

[ece36265-bib-0060] Seddon, P. J. (2010). From reintroduction to assisted colonization: Moving along the conservation translocation spectrum. Restoration Ecology, 18, 796–802. 10.1111/j.1526-100X.2010.00724.x

[ece36265-bib-0061] Selonen, V. , Hanski, I. K. , & Desrochers, A. (2007). Natal habitat‐biased dispersal in the Siberian flying squirrel. Proceedings of the Royal Society B: Biological Sciences, 274, 2063–2068. 10.1098/rspb.2007.0570 PMC2275184 17567559

[ece36265-bib-0062] Shaw, G. , & Livingstone, J. (1992). The pine marten: Its reintroduction and subsequent history in the Galloway Forest Park. Transactions of the Dumfries and Galloway Natural History and Antiquarian Society, 67, 1–7.

[ece36265-bib-0063] Sheehy, E. , O’Meara, D. B. , O’Reilly, C. , Smart, A. , & Lawton, C. (2014). A non‐invasive approach to determining pine marten abundance and predation. European Journal of Wildlife Research, 60, 223–236.

[ece36265-bib-0064] Shier, D. M. , & Swaisgood, R. R. (2011). Fitness costs of neighborhood disruption in translocations of a solitary mammal. Conservation Biology, 26, 116–123. 10.1111/j.1523-1739.2011.01748.x 21978094

[ece36265-bib-0065] Sidorovich, V. E. , Sidorovich, A. A. , & Krasko, D. A. (2010). Effect of felling on red fox (*Vulpes vulpes*) and pine marten (*Martes martes*) diets in transitional mixed forest in Belarus. Mammal Biology, 75, 399–411. 10.1016/j.mambio.2009.10.003

[ece36265-bib-0066] Sjoasen, T. (1997). Movements and Establishment of Reintroduced European Otters *Lutra lutra* . Journal of Applied Ecology, 34, 1070–1080.

[ece36265-bib-0067] Slough, B. G. (1989). Movements and habitat use by transplanted marten in the Yukon Territory. Journal of Wildlife Management, 53, 991–997.

[ece36265-bib-0068] Spinola, R. M. , Serfass, T. L. , & Brooks, R. P. (2018). Survival and post‐release movements of river otters translocated to Western New York. Northeastern Naturalist, 15, 13–25. https://www.jstor.org/stable/25177079

[ece36265-bib-0069] Stamps, J. (2001). Habitat selection by dispersers: Integrating proximate and ultimate approaches In Clobert J., Danchin E., Dhondt A., & Nichols J. (Eds.) Dispersal (pp. 230–242). Oxford, UK: Oxford University Press.

[ece36265-bib-0070] Stamps, J. , & Krishnan, V. V. (2005). Nonintuitive cue use in habitat selection. Ecology, 86, 2860–2867.

[ece36265-bib-0071] Stamps, J. A. , & Swaisgood, R. R. (2007). Someplace like home: Experience, habitat selection and conservation biology. Applied Animal Behavioural Science, 102, 392–409. 10.1016/j.applanim.2006.05.038

[ece36265-bib-0072] Steventon, J. D. , & Major, J. T. (1982). Marten use of habitat in a commercially clear‐cut forest. Journal of Wildlife Management, 46, 175–182. https://www.jstor.org/stable/3808420 .

[ece36265-bib-0073] Storch, I. , Lindström, E. , & Jounge, J. D. (1990). Diet and habitat selection of the pine marten in relation to competition with the red fox. Acta Theriologica, 35, 311–320.

[ece36265-bib-0074] Tolhurst, B. , Grogan, A. , Hughes, H. , & Scott, D. (2015). Effects of temporary captivity on ranging behaviour in urban red foxes (*Vulpes vulpes)* . Applied Animal Behavioural Science, 181, 182–190. 10.1016/j.applanim.2016.05.004

[ece36265-bib-0075] Toms, J. D. , & Lesperance, M. L. (2003). Piecewise regression: A tool for identifying ecological thresholds. Ecology, 84, 20134–22041. 10.1890/0012

[ece36265-bib-0076] Ventura, F. , Matthiopoulos, J. , & Jeglinski, J. W. E. (2019). Minimal overlap between areas of high conservation priority for endangered Galapagos pinnipeds and the conservation zone of the Galapagos Marine Reserve. Aquatic Conservation: Marine and Freshwater Ecosystems, 29, 115–126. 10.1002/aqc.2943

[ece36265-bib-0077] Virgós, E. , Zalewski, A. , Rosalino, L. M. , & Mergey, M. (2012). Habitat ecology of *Martes* species in Europe In Aubry K. B., Zielinski W. J., Proulx G., & Buskirk S. W. (Eds.), Biology and conservation of martens, sables, and fishers: A new synthesis (pp. 255–266). Ithaca, NY: Cornell University Press.

[ece36265-bib-0078] Ward, M. P. , & Schlossberg, S. (2004). Conspecific attraction and the conservation of territorial songbirds. Conservation Biology, 18, 519–525.

[ece36265-bib-0079] Warton, D. , & Aarts, G. (2013). Advancing our thinking in presence‐only and used‐available analysis. Journal of Animal Ecology, 82, 1125–1134. 10.1111/1365-2656.12071 23488567

[ece36265-bib-0080] Weber, D. , Roth, T. , Tesini, C. , & Thiel, D. (2018). Widespread distribution of Pine martens (*Martes martes*) in a fragmented suburban landscape. Mammal Research, 63, 349–356.

[ece36265-bib-0081] Woodford, J. E. , Macfarland, D. M. , & Worland, M. (2013). Movement, survival, and home range size of translocated American martens (*Martes americana*) in Wisconsin. Wildlife Society Bulletin, 37, 616–622. 10.1002/wsb.291

[ece36265-bib-0082] Ydenberg, R. C. , Giraldeau, L. A. , & Falls, J. B. (1988). Neighbours, strangers, and the asymmetric war of attrition. Animal Behaviour, 36, 343–347. 10.1016/S0003-3472(88)80004-6

[ece36265-bib-0083] Yott, A. , Rosatte, R. , Schaefer, J. A. , Hamr, J. , & Fryxell, J. (2011). Movement and spread of a founding population of reintroduced Elk (*Cervus elaphus*) in Ontario. Canadian Restoration Ecology, 19, 70–77. 10.1111/j.1526-100X.2009.00639.x

[ece36265-bib-0084] Zalewski, A. (1997). Patterns of resting site use by pine marten *Martes martes* in Białowieża National Park (Poland). Acta Theriologica, 42, 153–168.

[ece36265-bib-0085] Zalewski, A. (2007). Does size dimorphism reduce competition between sexes? The diet of male and female pine martens at local and wider geographical scales. Acta Theriologica, 52, 237–250.

[ece36265-bib-0086] Zuur, A. , Ieno, E. N. , Walker, N. , Saveliev, A. A. , & Smith, G. M. (2009). Mixed effects models and extensions in ecology with R. New York, NY: Springer Verlag

