# Supplementary material for: Postrelease movement and habitat selection of translocated pine martens Martes martes
Source: Ecol Evol. 2020 May 14;10(11):5106–18. doi: 10.1002/ece3.6265 (PMC7297779; doi:10.1002/ece3.6265)
Supplement: Supplementary file 1 — Supplementary Material [file ECE3-10-5106-s001.docx]

| **Broad habitat type** | **CORINE Level 3 Description** | **CORINE**  **Level 3 Code** |
| --- | --- | --- |
| Agricultural | Non-irrigated arable land | 211 |
|  | Land principally occupied by agriculture, with significant areas of natural vegetation | 243 |
|  | Pastures | 231 |
| Forest | Broad-leaved forest | 311 |
|  | Coniferous forest | 312 |
|  | Mixed forest | 313 |
| Grassland | Natural grasslands | 321 |
|  | Moors and heathland | 322 |
|  | Transitional woodland-shrub | 324 |
|  | Beaches, dunes, sands | 331 |
|  | Bare rocks | 332 |
|  | Sparsely vegetated areas | 333 |
| **Forest type** | **National Forestry Inventory description** |  |
| Broadleaved | Broadleaved |  |
|  | Mixed mainly broadleaved |  |
| Conifer | Conifer |  |
|  | Mixed mainly conifer |  |
| Felled | Felled |  |
|  | Windblow |  |
|  | Ground Prep |  |
| Open | Agricultural land |  |
|  | Bare area |  |
|  | Windfarm |  |
|  | Grassland |  |
|  | Open water |  |
| Undefined | Young trees |  |
|  | Assumed woodland |  |
|  | Low density |  |
|  | Shrub |  |
|  | Other vegetation |  |

**Appendix 1**. Broad habitat and forest types and groupings for marten habitat preference analysis.


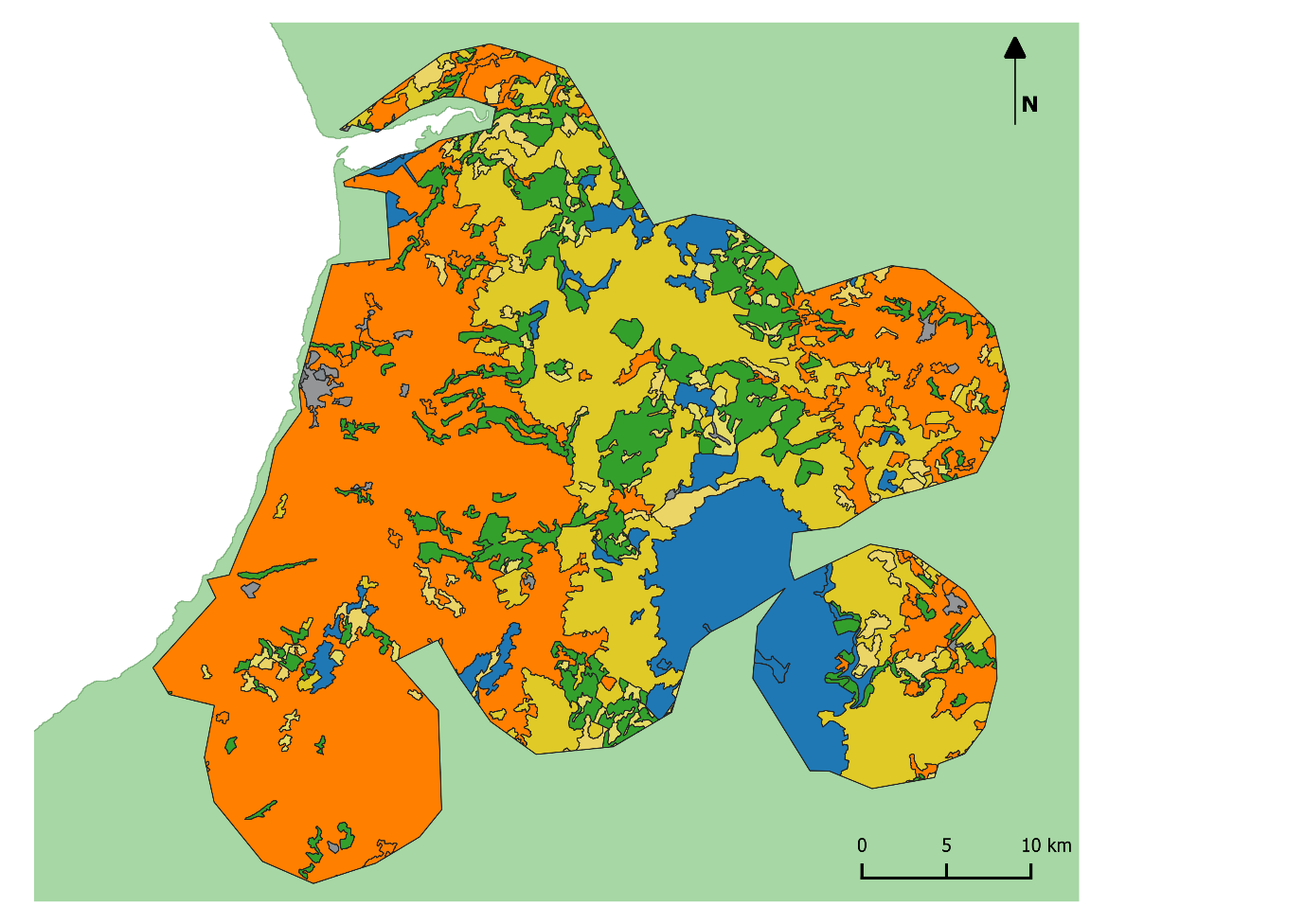


**Appendix 2.** Distribution of broad land use types within the study area. Study area is determined by marten locations and all land within 7.15 km of the centre of their home ranges. Land use types are shown by colour: Orange – agricultural land, yellow – grassland, green – forest, blue – wetland/bog, grey – urban areas. Details of specific land cover can be found in Appendix 1.

| **ID** | **Sex** | **Release date** | **Total no. of fixes in 100 days** | **Used in movement analysis?** | **No. of fixes used for range size analysis** | **Used in home range size analysis?** | **Home range (90% KDE) size (km^2^)** |
| --- | --- | --- | --- | --- | --- | --- | --- |
| 1 | M | 10/09/2015 | 11 | Y | NA | N | NA |
| 2 | F | 10/09/2015 | 54 | Y | 45 | Y | 8.23 |
| 3 | M | 17/09/2015 | 40 | Y | 27 | Y | 12.21 |
| 4 | F | 16/09/2015 | 41 | Y | 39 | Y | 24.54 |
| 5 | M | 27/09/2015 | 41 | Y | 36 | Y | 15.54 |
| 6 | F | 12/10/2015 | 37 | Y | 35 | Y | 2.64 |
| 7 | F | 14/10/2015 | 39 | Y | 32 | Y | 0.86 |
| 8 | F | 25/10/2015 | 25 | Y | 14 | Y | 1.29 |
| 9 | M | 25/10/2015 | 16 | Y | 14 | Y | 21.81 |
| 10 | F | 30/10/2015 | 21 | Y | 20 | Y | 3.28 |
| 11 | M | 30/10/2015 | 23 | N | 23 | Y | 14.62 |
| 12 | F | 08/11/2015 | 13 | N | 13 | Y | 4.53 |
| 13 | M | 15/11/2015 | 18 | Y | 16 | Y | 2.08 |
| 14 | F | 11/09/2016 | 49 | Y | 46 | Y | 3.19 |
| 15 | F | 10/09/2016 | 30 | Y | 22 | Y | 17.50 |
| 16 | M | 10/09/2016 | 74 | Y | 70 | Y | 10.86 |
| 17 | M | 11/09/2016 | 43 | Y | 41 | Y | 10.00 |
| 18 | F | 18/09/2016 | 14 | Y | 11 | Y | 17.46 |
| 19 | F | 17/09/2016 | 32 | Y | 28 | Y | 2.55 |
| 20 | M | 18/09/2016 | 47 | Y | 47 | Y | 1.82 |
| 21 | M | 25/09/2016 | 44 | Y | 41 | Y | 2.46 |
| 22 | F | 24/09/2016 | 12 | Y | NA | N | NA |
| 23 | M | 22/09/2016 | 83 | Y | 78 | Y | 2.85 |
| 24 | M | 25/09/2016 | 30 | N | 30 | Y | 6.51 |
| 25 | F | 02/10/2016 | 28 | N | 28 | Y | 6.45 |
| 26 | M | 01/10/2016 | 84 | Y | 82 | Y | 43.75 |
| 27 | M | 08/10/2016 | 46 | Y | 42 | Y | 7.85 |
| 28 | F | 08/10/2016 | 26 | Y | 21 | Y | 0.47 |
| 29 | M | 09/10/2016 | 7 | N | NA | N | NA |

**Appendix 3.** Details of individual pine martens, the number of fixes taken within 100 days and the analyses in which they were used.
